# Poly[μ_3_-β-alanine-aqua-μ_4_-sulfato-dilithium]

**DOI:** 10.1107/S1600536812002115

**Published:** 2012-01-25

**Authors:** M. Daniel Sweetlin, Shibu M. Eapen, S. Perumal, S. Ramalingom

**Affiliations:** a Physics Research Centre, S.T. Hindu College, Nagercoil 629 002, India; b Scientist in charge, SAIF, STIC, Cochin University of Science & Technology, Cochin 682 022, India; c Department of Physics, Vivekananda College, Agasteeswaram 629 701, India

## Abstract

The title compound, [Li_2_(SO_4_)(C_3_H_7_NO_2_)(H_2_O)]_*n*_, is a coordination polymer in which the β-alanine residues remain in the zwitterionic form. The crystal structure consists of corrugated sheets of [LiO_4_] and [SO_4_] tetra­hedra parallel to (010) with the β-alanine mol­ecules located between the sheets. The two independent Li^+^ cations are four-coordinated by O atoms in a distorted tetra­hedral geometry. The crystal structure is formed by stacking of alternate organic and inorganic layers along the *a* axis. The crystal structure is further stabilized by N—H⋯O hydrogen bonds.

## Related literature

For related structures with glycine as the amino acid, see: Fleck & Bohatý (2004 ▶). For related metal-organic compounds, see: Anbuchezhiyan *et al.* (2010 ▶); Liao *et al.* (2001 ▶); Pestov *et al.* (2005 ▶); Urpí *et al.* (2003 ▶). For the importance of β-alanine and lithium in medicine and pharmaceuticals, see: Anderson *et al.* (2008 ▶); Cipriani *et al.* (2005 ▶); Derave *et al.* (2007 ▶); Geddes *et al.* (2004 ▶); Poolsup *et al.* (2000 ▶); Tiedje *et al.* (2010 ▶).
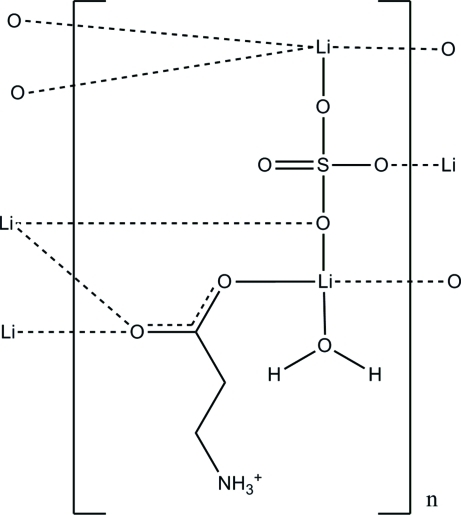


## Experimental

### 

#### Crystal data

[Li_2_(SO_4_)(C_3_H_7_NO_2_)(H_2_O)]
*M* *_r_* = 217.05
Triclinic, 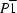

*a* = 5.1093 (4) Å
*b* = 9.2367 (8) Å
*c* = 9.6769 (8) Å
α = 68.725 (3)°
β = 82.576 (3)°
γ = 89.045 (3)°
*V* = 421.77 (6) Å^3^
*Z* = 2
Mo *K*α radiation
μ = 0.39 mm^−1^
*T* = 296 K
0.35 × 0.30 × 0.25 mm

#### Data collection

Bruker Kappa APEXII CCD diffractometer
Absorption correction: multi-scan (*SADABS*; Bruker, 1999 ▶) *T*_min_ = 0.875, *T*_max_ = 0.909
6764 measured reflections
2045 independent reflections
1899 reflections with *I* > 2σ(*I*)
*R*_int_ = 0.062

#### Refinement

*R*[*F* ^2^ > 2σ(*F* ^2^)] = 0.041
*wR*(*F* ^2^) = 0.115
*S* = 1.07
2045 reflections
163 parameters
3 restraints
H atoms treated by a mixture of independent and constrained refinement
Δρ_max_ = 0.42 e Å^−3^
Δρ_min_ = −0.50 e Å^−3^

### 

Data collection: *APEX2* (Bruker, 2004 ▶); cell refinement: *APEX2* and *SAINT* (Bruker, 2004 ▶); data reduction: *SAINT* and *XPREP* (Bruker, 2004 ▶); program(s) used to solve structure: *SIR92* (Altomare *et al.*, 1993 ▶); program(s) used to refine structure: *SHELXL97* (Sheldrick, 2008 ▶); molecular graphics: *OLEX2* (Dolomanov *et al.*, 2009 ▶); software used to prepare material for publication: *SHELXL97*, *PLATON* (Spek, 2009 ▶) and *publCIF* (Westrip, 2010 ▶).

## Supplementary Material

Crystal structure: contains datablock(s) I, global. DOI: 10.1107/S1600536812002115/zj2051sup1.cif

Structure factors: contains datablock(s) I. DOI: 10.1107/S1600536812002115/zj2051Isup2.hkl

Supplementary material file. DOI: 10.1107/S1600536812002115/zj2051Isup3.cml

Additional supplementary materials:  crystallographic information; 3D view; checkCIF report

## Figures and Tables

**Table 1:** Hydrogen-bond geometry (Å, °)

*D*—H⋯*A*	*D*—H	H⋯*A*	*D*⋯*A*	*D*—H⋯*A*
N—H*NA*⋯O5^i^	0.94 (4)	2.58 (4)	2.981 (2)	106 (3)
N—H*NA*⋯O4^i^	0.94 (4)	2.25 (4)	3.082 (3)	147 (3)
N—H*NB*⋯O4^ii^	0.88 (4)	2.02 (4)	2.851 (2)	157 (4)
N—H*NC*⋯O2	0.93 (3)	2.32 (3)	2.928 (2)	123 (2)
N—H*NC*⋯O2^iii^	0.93 (3)	2.12 (3)	2.947 (2)	148 (2)

Symmetry codes: (i) 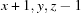; (ii) 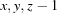; (iii) 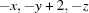.

**Fig. 1.**
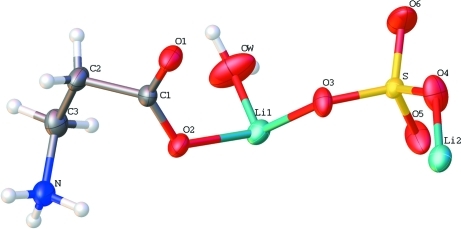
The asymmetric unit of the title compound, with atom labels and anisotropic displacement ellipsoids drawn at the 50% probability level.

**Fig. 2.**
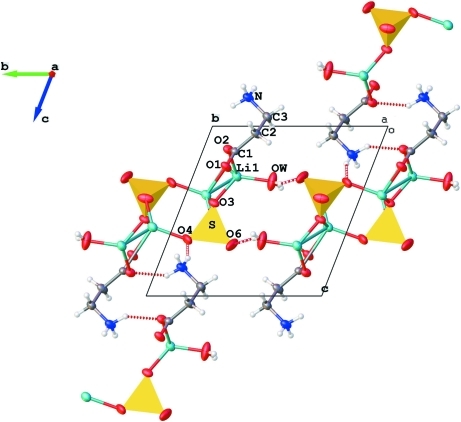
Molecular packing of the title compound as viewed down the crytallographic a axis. Hydrogen bonds are represented by red dotted lines.

## supplementary crystallographic information

### Comment

Naturally available β-alanine is constituent of the dipeptides, carnosine and anserine. It has the ability to form coordinate complexes with different metals both transition and nontransition elements due to its free carboxylate anion in its zwitterionic form. Previous reports have shown that β-alanine was forming crystalline complexes with organic and inorganic compounds (Liao *et al.*, 2001; Urpí *et al.*, 2003; Pestov *et al.*, 2005; Anbuchezhiyan *et al.*, 2010).

Herein, we are reporting a very interesting crystal structure of β-alanine with lithium sulfate. Both β-alanine and lithium got tremendous interest to chemists due to their importance in medicine and pharmaceuticals (Poolsup *et al.*, 2000; Cipriani *et al.*, 2005; Anderson *et al.*, 2008; Tiedje *et al.*, 2010). Recently β-alanine is gaining momentum as a sports medicine (Derave *et al.*, 2007) and Lithium remains as the 'gold standard' drug as mood stabiliser suitable for bipolar disorder (Geddes *et al.*,2 004). Hence the study of the title compound, which is formed by the combination of two potential drugs *viz*. β-alanine and lithium sulfate, will be very much useful for drug design and identification of the material.

The asymmetric unit (Fig.1) contains one-half of the compound, the other half being related to the first by an inversion centre. The structure of the title compound (Fig.2), is composed of corrugated sheets of [LiO_4_] tetrahedra and [SO_4_] tetrahedra parallel to (010). These sheets consist of three crystallographically different tetrahedra (around atoms Li1, Li2 and S). These tetrahedra are connected by common corners with O atoms. The tetrahedra around Li1, Li2 are connected by O1 and Li1, S by O3. The tetrahedron around S is connected with three Li2 tetrahedra by O3, O4 and O5. The tip of each tetrahedron faces away from the sheet. The coordination environment around the Li1 and Li2 atoms involving O atoms form distorted tetrahedron because the coordinating O atoms have dissimilar attachments. The Li1 atom is coordinated by two O atoms from two different β–alanine carboxyl anions, one O from the water ligand and another O from the SO_4_ ligand. The Li2 atom is also four-coordinated by four O atoms of which three O atoms are from SO_4_ group of different asymmetric units and another O is from the carboxyl anion of the β–alanine ligand. The tetrahedral environment around S atom is regular with tetrahedral angle 109.121 (75)° as all the four O atoms attached to it have similarity in their association with atoms on the other end by having coordination with either Li1 or Li2 atoms only.

### Experimental

All reagents were used as obtained commercially without further purification. A mixture containing β–alanine (89.1 mg, 1 mmol) and lithium sulfate monohydrate (127.9 mg, 1 mmol) were dissolved in 10 ml distilled water and heated to 50 °C for 2 h. The hot solution was filtered into a test tube and cooled to room temperature (30 °C). Colourless transparent crystals of the title compound were formed after four weeks which were suitable for single-crystal X-ray diffraction.

Primary characterization of the title compound was carried out by FTIR spectroscopy, Differential Scanning Calorimetry (DSC), Thermogravimetric Analysis (TGA) and CHNS elemental analysis. Following are the results of the CHNS elemental analysis for the tittle compound. Calculated: C, 16.60%; H, 4.19%; N, 6.45%; S, 14.77%. Observed: C, 16.87%; H, 3.57%; N, 6.48%; S, 12.4%. The close agreement between the calculated and observed values shows that the molecules of β-alanine, lithium sulfate and water have combined in equimolar ratio to form the title compound. From TGA we observed a weight loss of 8% between 166°C and 193°C which shows the presence of water molecules in the equimolar ratio in the title compound.

### Refinement

The water H atoms were located in a difference Fourier, and refined isotropically with O—H restraints (0.86 (2) Å). All other H atoms were positioned geometrically (C—H = 0.96–0.97 Å; N—H = 0.91 Å) and in the refinement process were allowed to ride on their carrier atoms with *U*iso(H) = 1.2*U*eq(C, N).

### Crystal data

**Table:**

[Li_2_(SO_4_)(C_3_H_7_NO_2_)(H_2_O)]	*Z* = 2
*M**_r_* = 217.05	*F*(000) = 224
Triclinic, *P*1	*D*_x_ = 1.709 Mg m^−^^3^*D*_m_ = 1.71 Mg m^−^^3^*D*_m_ measured by Floatation
Hall symbol: -P 1	Melting point: 457.9 K
*a* = 5.1093 (4) Å	Mo *K*α radiation, λ = 0.71073 Å
*b* = 9.2367 (8) Å	Cell parameters from 184 reflections
*c* = 9.6769 (8) Å	θ = 2.3–24.3°
α = 68.725 (3)°	µ = 0.39 mm^−^^1^
β = 82.576 (3)°	*T* = 296 K
γ = 89.045 (3)°	Block, colourless
*V* = 421.77 (6) Å^3^	0.35 × 0.30 × 0.25 mm

### Data collection

**Table:**

Bruker Kappa APEXII CCD diffractometer	2045 independent reflections
Radiation source: fine-focus sealed tube	1899 reflections with *I* > 2σ(*I*)
graphite	*R*_int_ = 0.062
ω and φ scan	θ_max_ = 28.3°, θ_min_ = 2.6°
Absorption correction: multi-scan (*SADABS*; Bruker, 1999)	*h* = −6→6
*T*_min_ = 0.875, *T*_max_ = 0.909	*k* = −12→12
6764 measured reflections	*l* = −12→12

### Refinement

**Table:**

Refinement on *F*^2^	Primary atom site location: structure-invariant direct methods
Least-squares matrix: full	Secondary atom site location: difference Fourier map
*R*[*F*^2^ > 2σ(*F*^2^)] = 0.041	Hydrogen site location: inferred from neighbouring sites
*wR*(*F*^2^) = 0.115	H atoms treated by a mixture of independent and constrained refinement
*S* = 1.07	*w* = 1/[σ^2^(*F*_o_^2^) + (0.0528*P*)^2^ + 0.2641*P*] where *P* = (*F*_o_^2^ + 2*F*_c_^2^)/3
2045 reflections	(Δ/σ)_max_ = 0.001
163 parameters	Δρ_max_ = 0.42 e Å^−^^3^
3 restraints	Δρ_min_ = −0.50 e Å^−^^3^

### Special details

**Table:**

Geometry. All s.u.'s (except the s.u. in the dihedral angle between two l.s. planes) are estimated using the full covariance matrix. The cell s.u.'s are taken into account individually in the estimation of s.u.'s in distances, angles and torsion angles; correlations between s.u.'s in cell parameters are only used when they are defined by crystal symmetry. An approximate (isotropic) treatment of cell s.u.'s is used for estimating s.u.'s involving l.s. planes.
Refinement. Refinement of *F*^2^ against ALL reflections. The weighted *R*-factor *wR* and goodness of fit *S* are based on *F*^2^, conventional *R*-factors *R* are based on *F*, with *F* set to zero for negative *F*^2^. The threshold expression of *F*^2^ > 2σ(*F*^2^) is used only for calculating *R*-factors(gt) *etc*. and is not relevant to the choice of reflections for refinement. *R*-factors based on *F*^2^ are statistically about twice as large as those based on *F*, and *R*- factors based on ALL data will be even larger.

### Fractional atomic coordinates and isotropic or equivalent isotropic displacement parameters (Å^2^)

**Table:**

	*x*	*y*	*z*	*U*_iso_*/*U*_eq_	
S	−0.30781 (7)	0.77817 (4)	0.59785 (4)	0.01721 (16)	
O5	−0.5839 (3)	0.7797 (2)	0.57385 (16)	0.0396 (4)	
O4	−0.2641 (3)	0.89203 (17)	0.66688 (16)	0.0356 (4)	
O2	0.0637 (2)	0.85784 (15)	0.13776 (14)	0.0254 (3)	
O3	−0.1386 (2)	0.82127 (16)	0.45218 (14)	0.0261 (3)	
O1	0.4349 (2)	0.83197 (17)	0.23942 (15)	0.0287 (3)	
O6	−0.2486 (4)	0.62525 (19)	0.69837 (19)	0.0535 (5)	
C1	0.2965 (3)	0.81463 (19)	0.14778 (18)	0.0197 (3)	
C2	0.4192 (4)	0.7346 (3)	0.0438 (2)	0.0291 (4)	
C3	0.2349 (4)	0.7090 (2)	−0.0549 (2)	0.0287 (4)	
N	0.1701 (4)	0.8593 (2)	−0.16740 (19)	0.0304 (4)	
OW	−0.1998 (4)	0.53707 (19)	0.3258 (3)	0.0581 (6)	
Li2	−0.2349 (6)	1.1171 (4)	0.5940 (3)	0.0261 (6)	
Li1	−0.2272 (6)	0.7488 (4)	0.2953 (4)	0.0256 (6)	
HNA	0.321 (7)	0.912 (4)	−0.232 (4)	0.060 (9)*	
HNB	0.055 (7)	0.846 (4)	−0.222 (4)	0.069 (10)*	
HWB	−0.325 (5)	0.473 (3)	0.364 (4)	0.069 (10)*	
H3A	0.321 (5)	0.649 (3)	−0.109 (3)	0.040 (7)*	
H3B	0.070 (6)	0.660 (3)	0.004 (3)	0.047 (7)*	
H4B	0.482 (6)	0.643 (4)	0.097 (3)	0.052 (8)*	
H4A	0.569 (6)	0.793 (3)	−0.013 (3)	0.054 (8)*	
HWA	−0.058 (5)	0.485 (4)	0.340 (5)	0.107 (15)*	
HNC	0.109 (5)	0.927 (3)	−0.120 (3)	0.033 (6)*	

### Atomic displacement parameters (Å^2^)

**Table:**

	*U*^11^	*U*^22^	*U*^33^	*U*^12^	*U*^13^	*U*^23^
S	0.0138 (2)	0.0201 (2)	0.0187 (2)	0.00037 (14)	−0.00144 (14)	−0.00835 (16)
O5	0.0141 (6)	0.0760 (11)	0.0284 (7)	−0.0078 (6)	−0.0020 (5)	−0.0183 (7)
O4	0.0448 (8)	0.0376 (8)	0.0311 (7)	−0.0141 (6)	0.0027 (6)	−0.0222 (6)
O2	0.0183 (6)	0.0344 (7)	0.0255 (6)	0.0063 (5)	−0.0024 (5)	−0.0136 (5)
O3	0.0172 (6)	0.0399 (8)	0.0233 (6)	−0.0055 (5)	0.0032 (4)	−0.0156 (5)
O1	0.0231 (6)	0.0419 (8)	0.0303 (7)	0.0084 (5)	−0.0090 (5)	−0.0224 (6)
O6	0.0649 (12)	0.0302 (8)	0.0444 (9)	0.0221 (8)	0.0117 (8)	0.0039 (7)
C1	0.0185 (7)	0.0226 (8)	0.0175 (7)	0.0024 (6)	−0.0004 (5)	−0.0073 (6)
C2	0.0266 (9)	0.0402 (10)	0.0275 (9)	0.0141 (8)	−0.0067 (7)	−0.0200 (8)
C3	0.0341 (10)	0.0304 (9)	0.0260 (9)	0.0004 (7)	−0.0028 (7)	−0.0157 (7)
N	0.0311 (8)	0.0404 (9)	0.0245 (8)	0.0098 (7)	−0.0064 (7)	−0.0168 (7)
OW	0.0394 (10)	0.0254 (8)	0.1070 (17)	0.0012 (7)	−0.0107 (10)	−0.0209 (9)
Li2	0.0187 (13)	0.0341 (16)	0.0290 (15)	−0.0015 (11)	−0.0018 (11)	−0.0161 (13)
Li1	0.0190 (13)	0.0311 (16)	0.0308 (15)	0.0034 (11)	−0.0042 (11)	−0.0160 (13)

### Geometric parameters (Å, °)

**Table:**

S—O6	1.4484 (15)	C3—N	1.485 (3)
S—O5	1.4579 (13)	C3—H3A	0.96 (3)
S—O4	1.4692 (13)	C3—H3B	0.97 (3)
S—O3	1.4772 (12)	N—HNA	0.94 (3)
O5—Li2^i^	1.908 (4)	N—HNB	0.88 (4)
O4—Li2	1.939 (4)	N—HNC	0.93 (3)
O2—C1	1.253 (2)	OW—Li1	1.875 (4)
O2—Li1	1.974 (3)	OW—HWB	0.833 (18)
O3—Li2^ii^	1.948 (3)	OW—HWA	0.859 (19)
O3—Li1	1.970 (3)	Li2—O5^i^	1.908 (4)
O1—C1	1.257 (2)	Li2—O3^ii^	1.948 (3)
O1—Li1^iii^	1.939 (3)	Li2—O1^ii^	1.994 (3)
O1—Li2^ii^	1.994 (3)	Li2—C1^ii^	2.771 (3)
C1—C2	1.521 (2)	Li2—Li1^ii^	3.157 (4)
C1—Li2^ii^	2.771 (3)	Li2—Li1^i^	3.214 (4)
C2—C3	1.503 (3)	Li1—O1^iv^	1.939 (3)
C2—H4B	0.90 (3)	Li1—Li2^ii^	3.157 (4)
C2—H4A	0.93 (3)	Li1—Li2^i^	3.214 (4)
			
O6—S—O5	109.41 (11)	Li1—OW—HWB	123 (2)
O6—S—O4	108.78 (11)	Li1—OW—HWA	125 (3)
O5—S—O4	108.91 (10)	HWB—OW—HWA	106 (3)
O6—S—O3	111.20 (9)	O5^i^—Li2—O4	114.67 (17)
O5—S—O3	109.12 (8)	O5^i^—Li2—O3^ii^	110.71 (16)
O4—S—O3	109.39 (8)	O4—Li2—O3^ii^	108.30 (16)
S—O5—Li2^i^	133.35 (13)	O5^i^—Li2—O1^ii^	104.37 (15)
S—O4—Li2	134.19 (13)	O4—Li2—O1^ii^	103.06 (15)
C1—O2—Li1	120.86 (14)	O3^ii^—Li2—O1^ii^	115.69 (16)
S—O3—Li2^ii^	128.04 (12)	O5^i^—Li2—C1^ii^	123.53 (15)
S—O3—Li1	121.15 (11)	O4—Li2—C1^ii^	103.92 (14)
Li2^ii^—O3—Li1	107.36 (14)	O3^ii^—Li2—C1^ii^	93.11 (12)
C1—O1—Li1^iii^	131.16 (15)	O1^ii^—Li2—C1^ii^	24.28 (6)
C1—O1—Li2^ii^	115.02 (14)	O5^i^—Li2—Li1^ii^	128.01 (16)
Li1^iii^—O1—Li2^ii^	109.61 (14)	O4—Li2—Li1^ii^	114.65 (15)
O2—C1—O1	124.14 (15)	O3^ii^—Li2—Li1^ii^	36.56 (9)
O2—C1—C2	118.10 (15)	O1^ii^—Li2—Li1^ii^	79.45 (12)
O1—C1—C2	117.76 (15)	C1^ii^—Li2—Li1^ii^	56.56 (9)
O2—C1—Li2^ii^	84.56 (11)	O5^i^—Li2—Li1^i^	70.83 (11)
O1—C1—Li2^ii^	40.69 (10)	O4—Li2—Li1^i^	109.49 (14)
C2—C1—Li2^ii^	155.18 (14)	O3^ii^—Li2—Li1^i^	136.76 (16)
C3—C2—C1	114.33 (15)	O1^ii^—Li2—Li1^i^	34.63 (8)
C3—C2—H4B	109.1 (18)	C1^ii^—Li2—Li1^i^	57.93 (9)
C1—C2—H4B	109.9 (19)	Li1^ii^—Li2—Li1^i^	106.63 (13)
C3—C2—H4A	110.9 (19)	OW—Li1—O1^iv^	113.60 (17)
C1—C2—H4A	108.1 (19)	OW—Li1—O3	118.66 (18)
H4B—C2—H4A	104 (3)	O1^iv^—Li1—O3	107.99 (15)
N—C3—C2	110.69 (17)	OW—Li1—O2	106.31 (16)
N—C3—H3A	107.1 (15)	O1^iv^—Li1—O2	110.88 (16)
C2—C3—H3A	109.1 (16)	O3—Li1—O2	98.23 (14)
N—C3—H3B	107.3 (16)	OW—Li1—Li2^ii^	113.59 (15)
C2—C3—H3B	110.7 (16)	O1^iv^—Li1—Li2^ii^	131.51 (16)
H3A—C3—H3B	112 (2)	O3—Li1—Li2^ii^	36.08 (8)
C3—N—HNA	112.0 (19)	O2—Li1—Li2^ii^	64.94 (11)
C3—N—HNB	111 (2)	OW—Li1—Li2^i^	121.10 (16)
HNA—N—HNB	108 (3)	O1^iv^—Li1—Li2^i^	35.76 (9)
C3—N—HNC	110.0 (15)	O3—Li1—Li2^i^	74.83 (11)
HNA—N—HNC	104 (2)	O2—Li1—Li2^i^	129.47 (15)
HNB—N—HNC	111 (3)	Li2^ii^—Li1—Li2^i^	106.63 (13)
			
O6—S—O5—Li2^i^	−145.5 (2)	Li2^ii^—C1—C2—C3	150.7 (3)
O4—S—O5—Li2^i^	95.8 (2)	C1—C2—C3—N	68.4 (2)
O3—S—O5—Li2^i^	−23.6 (2)	S—O4—Li2—O5^i^	26.6 (3)
O6—S—O4—Li2	166.47 (18)	S—O4—Li2—O3^ii^	−97.5 (2)
O5—S—O4—Li2	−74.36 (19)	S—O4—Li2—O1^ii^	139.41 (15)
O3—S—O4—Li2	44.8 (2)	S—O4—Li2—C1^ii^	164.38 (13)
O6—S—O3—Li2^ii^	−73.80 (19)	S—O4—Li2—Li1^ii^	−136.35 (15)
O5—S—O3—Li2^ii^	165.42 (16)	S—O4—Li2—Li1^i^	103.89 (18)
O4—S—O3—Li2^ii^	46.37 (18)	S—O3—Li1—OW	−69.2 (2)
O6—S—O3—Li1	82.47 (17)	Li2^ii^—O3—Li1—OW	91.4 (2)
O5—S—O3—Li1	−38.31 (16)	S—O3—Li1—O1^iv^	61.9 (2)
O4—S—O3—Li1	−157.36 (14)	Li2^ii^—O3—Li1—O1^iv^	−137.53 (16)
Li1—O2—C1—O1	−71.1 (2)	S—O3—Li1—O2	177.06 (10)
Li1—O2—C1—C2	108.25 (19)	Li2^ii^—O3—Li1—O2	−22.34 (18)
Li1—O2—C1—Li2^ii^	−61.09 (16)	S—O3—Li1—Li2^ii^	−160.61 (19)
Li1^iii^—O1—C1—O2	169.51 (18)	S—O3—Li1—Li2^i^	48.28 (13)
Li2^ii^—O1—C1—O2	15.4 (2)	Li2^ii^—O3—Li1—Li2^i^	−151.12 (17)
Li1^iii^—O1—C1—C2	−9.8 (3)	C1—O2—Li1—OW	−51.3 (2)
Li2^ii^—O1—C1—C2	−163.97 (17)	C1—O2—Li1—O1^iv^	−175.22 (15)
Li1^iii^—O1—C1—Li2^ii^	154.1 (3)	C1—O2—Li1—O3	71.88 (19)
O2—C1—C2—C3	−3.3 (3)	C1—O2—Li1—Li2^ii^	57.57 (16)
O1—C1—C2—C3	176.12 (17)	C1—O2—Li1—Li2^i^	148.96 (17)

Symmetry codes: (i) −*x*−1, −*y*+2, −*z*+1; (ii) −*x*, −*y*+2, −*z*+1; (iii) *x*+1, *y*, *z*; (iv) *x*−1, *y*, *z*.

### Hydrogen-bond geometry (Å, °)

**Table:**

*D*—H···*A*	*D*—H	H···*A*	*D*···*A*	*D*—H···*A*
N—HNA···O5^v^	0.94 (4)	2.58 (4)	2.981 (2)	106 (3)
N—HNA···O4^v^	0.94 (4)	2.25 (4)	3.082 (3)	147 (3)
N—HNB···O4^vi^	0.88 (4)	2.02 (4)	2.851 (2)	157 (4)
N—HNC···O2	0.93 (3)	2.32 (3)	2.928 (2)	123 (2)
N—HNC···O2^vii^	0.93 (3)	2.12 (3)	2.947 (2)	148 (2)

Symmetry codes: (v) *x*+1, *y*, *z*−1; (vi) *x*, *y*, *z*−1; (vii) −*x*, −*y*+2, −*z*.
